# Adapting an Intervention to Address Barriers to Pain Management in Hospice: Formative Research to Inform EMPOWER-D for Dementia Caregivers

**DOI:** 10.1089/pmr.2024.0024

**Published:** 2024-07-13

**Authors:** Karla T. Washington, Morgan L. Van Vleck, Todd D. Becker, George Demiris, Debra Parker Oliver, Paul E. Tatum, Jacquelyn J. Benson, John G. Cagle

**Affiliations:** ^1^ Division of Palliative Medicine, Department of Medicine, Washington University School of Medicine in St. Louis, St. Louis, Missouri, USA.; ^2^ The Brown School at Washington University in St. Louis, St. Louis, Missouri, USA.; ^3^ Department of Surgery, Division of Public Health Sciences, Washington University School of Medicine in St. Louis, St. Louis, Missouri, USA.; ^4^ Department of Biobehavioral Health Sciences, University of Pennsylvania School of Nursing, Philadelphia, Pennsylvania, USA.; ^5^ Department of Biostatistics, Epidemiology and Informatics, University of Pennsylvania Perelman School of Medicine, Philadelphia, Pennsylvania, USA.; ^6^ Goldfarb School of Nursing at Barnes-Jewish College, St. Louis, Missouri, USA.; ^7^ Department of Veterans Affairs, VA St. Louis Health Care System, St. Louis, Missouri, USA.; ^8^ University of Maryland School of Social Work, Baltimore, Maryland, USA.

**Keywords:** caregivers, dementia, education, family, hospice, pain

## Abstract

**Background:**

Nearly half of more than 1.7 million older Americans who receive hospice care each year have a primary or comorbid diagnosis of dementia. Pain is often undertreated in this patient population owing to myriad factors, including unmet informational needs among family caregivers.

**Objective:**

We sought to inform the adaptation of a pain education intervention for hospice family caregivers to the context of dementia by eliciting feedback on the educational content covered in adapted intervention materials.

**Design:**

We conducted a multimethod, formative research study to inform the adaptation of an existing, evidence-based intervention.

**Setting/Subjects:**

The study included a purposively recruited sample (*n* = 33) of hospice professionals (*n* = 18) and family caregivers (*n* = 15) from across the United States.

**Measurements:**

Participants quantitatively rated the importance of each of the eight pain concerns presented in the adapted intervention materials (1 = not important to 3 = very important) and provided qualitative feedback via Zoom interview on the acceptability, clinical accuracy, and potential benefits of the adapted content. We analyzed quantitative data via descriptive statistics and qualitative data via content analysis.

**Results:**

Participants rated the adapted educational content as highly important (range_mean_ = 2.56–3.00), particularly regarding concerns about caregivers’ pain assessment, communicating with the hospice team about pain, and addressing misinformation regarding pain medication outcomes. Participants also provided suggestions to strengthen specific educational messages to improve comprehension and uptake.

**Conclusions:**

Findings support the continued development and testing of the adapted intervention.

## Introduction

Hospice care focuses on reducing suffering and promoting quality of life for individuals with serious and life-limiting illnesses.^1^ More than 1.7 million older Americans receive hospice services each year.^2^ Nearly half have a primary or comorbid diagnosis of Alzheimer’s disease or a related dementia (ADRD).^3^ Increased aging of the US demographic, alongside sustained growth in hospice utilization and rising ADRD prevalence, emphasizes the need to develop care practices for older hospice patients with ADRD.^4,5^

Pain management is a priority for hospice patients, including those with ADRD, most (63%) of whom experience bothersome pain.^6^ Barriers to optimal pain management in hospice care are well-known and have been the focus of numerous interventions.^7–15^ One such intervention, EMPOWER (Effective Management of Pain: Overcoming Worries to Enable Relief), has been shown to improve hospice pain management by training hospice staff on barriers to pain management, incorporating screening for pain concerns into routine hospice care, delivering tailored pain education to hospice patients and their family caregivers, and facilitating needed follow-up services.^16^ Noted outcomes shown to be positively affected by EMPOWER include hospice patient pain and hospice family caregivers’ concerns about pain and pain medications.^16^

From 2021 to 2022, we conducted a feasibility study of a remotely delivered version of EMPOWER for rural hospice family caregivers.^17^ Nearly all participants found the intervention acceptable as delivered, but several also recommended modifications. Family caregivers of patients with ADRD, in particular, suggested challenges associated with managing pain amid patients’ cognitive and functional decline as areas of needed support.^17^

These family caregivers’ suggestions reflect ongoing efforts to enhance pain management for hospice patients with dementia.^18,19^ Symptoms of dementia (e.g., cognitive impairment, communication challenges) threaten patients’ ability to effectively report their pain.^20^ Absence of the gold standard of self-reporting pain places higher emphasis on successful—often nonverbal—assessment by proxy observation.^20,21^ Yet the high subjectivity of pain, contrasted with the low validity and reliability of observational pain assessment, renders proxy observation challenging.^20,21^ These challenges are exacerbated for family caregivers, who typically have less clinical training than health care professionals. Thus, these considerations highlight the need to develop pain assessment and management practices among family caregivers of hospice patients with dementia.

Accordingly, we convened an expert panel of geriatricians and hospice leaders to draft educational materials for an adapted version of EMPOWER addressing barriers to pain management for family caregivers of hospice patients with ADRD (Effective Management of Pain: Overcoming Worries to Enable Relief—Dementia [EMPOWER-D]). Notable adaptations stemming from this hour-long discussion included reordering content to reflect greater or lesser relevance of specific educational content, adding content on pain assessment challenges, and including content appropriate for home and nursing home settings (original and adapted content is provided in Table 1).

**Table 1.** Original (EMPOWER) and Modified (EMPOWER-D) Pain Concerns and Educational Messages

EMPOWER	EMPOWER-D
1. ADDICTION: “I don’t want to get addicted.” When pain medications are used to treat pain, addiction is highly unlikely. It is common for patients to require an increase in pain medication over time. This is not addiction. It is simply increased tolerance meaning that more is needed for the same effect.	1. I don’t want my family member to get addicted to pain medications. When medications are used as prescribed to treat pain in hospice, addiction is unlikely for those who have not had prior substance-use disorders. It is common for patients to require an increase in pain medication over time. This is not addiction. It is increased tolerance, meaning that more medication is needed for the same effect.
2. TOLERANCE: “I don’t want to build up a tolerance.” It is normal for your body to adjust to the pain medication. Your dose can be increased if necessary so the medication keeps working. Using medication now will not prevent it from working in the future.	2. I can’t tell if my family member is in pain. People with dementia can feel pain, but they often have difficulty communicating about pain. Your hospice team can teach you how to recognize signs that your family member may be experiencing pain, even if your family member is unable to speak.
3. SIDE EFFECTS: “I don’t want to be knocked out/tired/constipated.” The tiredness that is associated with pain medications usually disappears after a few days. If it doesn’t, the dose can be readjusted. Constipation can be treated with laxatives and stool softeners.	3. I’m worried about potential side effects of pain medications, like drowsiness and constipation. The tiredness that is associated with pain medications usually disappears after a few days. If it doesn’t, the dose can be adjusted. Laxatives can be used to prevent and treat constipation.
4. DENYING PAIN: “I don’t want people to think I’m weak or a whiner.” Pain can severely affect quality of life, so it is not a sign of weakness to admit to pain. Honest communication is essential for good pain management. Denying pain makes it more difficult to manage comfort.	4. Pain medications are being used to treat symptoms other than pain, and I don’t understand why. Some medications used to treat pain can also help with other symptoms like shortness of breath, which patients with dementia may experience near the end of life. For patients with dementia, behaviors like moaning, crying, or hitting may be signs of pain. These behaviors may decrease when a person’s pain is treated.
5. DRUG-SEEKING: “I don’t want to appear like I’m drug seeking or a junkie.” Pain medication is appropriate for patients who are in pain. When someone on hospice says they are in pain, we believe them. Nearly 90% of patients receiving hospice care take pain medicine.	5. I don’t want my hospice team to think I am misusing my family member’s pain medications. Pain medication is appropriate for patients who are in pain; nearly 90% of hospice patients are prescribed pain medication. Your hospice team will show you how to safely store and keep track of pain medications.
6. BEING A BOTHER: “I don’t want to bother others.” Pain management is important and a key aspect of hospice care. If your pain is not being controlled well, we want you to call us day or night. If your current pain control treatments aren’t working, call us at [phone number]. We do not expect you to deal with your pain alone.	6. I don’t want to bother my hospice team or nursing home facility staff with pain management concerns. Pain management is an important focus of hospice care. If your family member’s pain is not well controlled, your hospice team wants to know. If your family member lives in a nursing home or other facility, facility staff members may play an important role in managing your family member’s pain. It may be helpful to talk about your family member’s pain with your hospice team and the facility staff members together.
7. TAKING TOO MUCH: “I’ll end up giving/taking too much.” Overdosing on pain medication is extremely rare. The hospice staff will provide ongoing assessments to see how you are responding to the medications. The dose can be adjusted if needed. If you stop giving/taking the pain medication, the pain will likely return and may be more difficult to get back under control.	7. I’m worried that pain medications will speed up the dying process. There is no evidence that pain medications speed up the dying process when a patient receives the right amount of medication to control their symptoms. Your hospice team will assess your family member’s symptoms on an ongoing basis and help you know how much medication should be given. If you have questions about how much medication should be given, you can call hospice any time—day or night.
8. TREATMENTS WON’T WORK: “Pain can’t be avoided” or “Only medications can treat pain.” Pain is not inevitable. Research tells us that most pain can be treated and comfort achieved. Non-drug treatments, such as massage, aroma therapy, and others, can also help to reduce pain. These nonmedication treatments can be used along with pain medications.	8. Other than using medications, I’m not sure how to help my family member feel better. Non-medication treatments like massage, application of warm or cool materials, moving a person’s body to a new position, or even introducing a pleasant experience like a nice smell or a favorite song may help your family member feel more comfortable. Your hospice team can help you select nonmedication treatments for your family member’s pain.

Extending these efforts, the objective of this study was to elicit feedback from hospice professionals and family caregivers on the educational content covered in these adapted intervention materials. Two questions guided our research:

1. How do hospice professionals and family caregivers rate the importance of the topics covered in EMPOWER-D?
2. How do hospice professionals and family caregivers describe the acceptability of EMPOWER-D’s educational content?

## Materials and Methods

We conducted a predominantly qualitative, multimethod, formative research study to answer our research questions and, thus, inform our intervention adaptation. Approval of research activities was granted by the Washington University in St. Louis and University of Pennsylvania Institutional Review Boards (#202204113 and #828990, respectively).

### Participant recruitment

#### Hospice professionals

Hospice professionals were recruited via social media and email outreach by professional organizations, targeted emails to prior research partners, and presentations to hospice agencies with which we had existing partnerships. Hospice professionals were eligible for study inclusion if they were (1) 18 years of age or older, (2) could speak and read English, and (3) were employed or affiliated with a Medicare-certified US hospice agency. Those interested in study participation received contact information for our study coordinator, who screened for eligibility, obtained verbal informed consent, and coordinated all subsequent research activities, including compensation of $50.

#### Hospice family caregivers

We employed purposive sampling^22^ from within an existing sample of dementia FCGs exiting a clinical trial of a supportive intervention for hospice family caregivers (R01NR012213; ClinicalTrials.gov ID: NCT03712410) to maximize sociodemographic variation for the current study. Family caregivers were eligible for study inclusion if they were (1) 18 years of age or older, (2) could speak and read English, and (3) were current or former family caregivers of a patient with ADRD receiving services from a Medicare-certified US hospice agency. After securing written informed consent, participating family caregivers were invited to provide feedback on the EMPOWER-D materials as part of their clinical trial exit interview. Family caregiver participants received $50 as compensation.

### Data collection and measures

Participants completed an approximately 30-minute, audio-recorded interview conducted via Zoom. During the interview, participants viewed slides individually listing eight common pain concerns and their corresponding messages comprising the EMPOWER-D educational materials (see Table 1). After reading each concern, participants were asked to (1) rate the importance of addressing the concern using a researcher-constructed Likert-type item (1 = *not important*, 2 = *somewhat important*, 3 = *very important*) and explain their rating, (2) discuss the acceptability of including the concern and the materials as written, (3) suggest changes to enhance the acceptability of the content, and (4) suggest modifications or additions to improve the clinical accuracy or potential benefits of the content (asked only of hospice professionals).

### Data analysis

After calculating descriptive statistics of quantitative data using Microsoft Excel, K.T.W. and M.L.V. performed a content analysis^23^ of transcribed qualitative data using NVivo. Content analysis entailed (1) reviewing the full qualitative dataset, (2) independently applying inductively derived manifest codes to excerpts that characterized participants’ perspectives on the importance and acceptability of each concern and its corresponding messages, (3) grouping similar codes into categories, and (4) discussing independently identified codes and categories to refine prospective findings. All discrepancies were minimal (e.g., code or category names) and reconciled via consensus-based discussion, thereby generating the final results. Quantitative and qualitative results were integrated during the interpretation of findings, providing a multifaceted depiction of participants’ feedback.^24^

## Results

### Quantitative

Participants (*n* = 33; hospice professionals: *n* = 18, family caregivers: *n* = 15; see Table 2) rated the importance of the EMPOWER-D pain concerns and corresponding educational messages as high, with mean importance ratings at or above 2.56 for all pain concerns (see Table 3). Family caregivers’ and hospice professionals’ ratings were most similar regarding the importance of addressing family caregivers’ lack of understanding about the use of pain medications to treat nonpain symptoms (which family caregivers rated as slightly more important than hospice professionals; mean difference = 0.08) and least similar regarding the importance of addressing family caregivers’ concerns about bothering others with pain concerns (which all family caregivers rated *very important*; mean difference = 0.33).

**Table 2.** Participant Characteristics (*n* = 33)

**Characteristic**	Hospice professionals (*n* = 18)	Family caregivers (*n* = 15)
Age range, *n* (%)		
18–29 years	2 (11%)	1 (7%)
30–39 years	4 (22%)	0 (0%)
40–49 years	4 (22%)	1 (7%)
50–59 years	5 (28%)	2 (13%)
60–69 years	3 (17%)	7 (47%)
70 years or older	0 (0%)	4 (27%)
Gender, *n* (%)		
Man	5 (28%)	2 (13%)
Woman	13 (72%)	13 (87%)
Race, *n* (%)		
Black	0 (0%)	6 (40%)
White	18 (100%)	6 (40%)
Multiracial	0 (0%)	2 (13%)
Declined to answer	0 (0%)	1 (7%)
Ethnicity, *n* (%)		
Hispanic	1 (6%)	2 (13%)
Non-Hispanic	17 (94%)	13 (87%)
Relationship to patient, *n* (%)		
Spouse/Partner	—	2 (13%)
Adult child/stepchild	—	12 (80%)
Grandchild	—	1 (7%)
Highest formal education, *n* (%)		
High school diploma or equivalent		3 (20%)
Some college/Trade school	—	2 (13%)
Associate’s degree	—	2 (13%)
Bachelor’s degree	—	3 (20%)
Graduate/Professional degree	—	5 (33%)
Profession, *n* (%)		
Chaplain	5 (28%)	—
Nurse	5 (28%)	—
Physician	3 (17%)	—
Social worker	5 (28%)	—
Professional experience, *n* (%)		
0–5 years	5 (28%)	—
6–10 years	4 (22%)	—
11–15 years	2 (11%)	—
16–20 years	1 (6%)	—
21–25 years	1 (6%)	—
More than 25 years	5 (28%)	—

Percentages may not equal 100% due to rounding.

**Table 3.** EMPOWER-D Pain Concern Importance Ratings and Participant Quotations (*n* = 33)

EMPOWER-D pain concern	EMPOWER-D importance ratings, mean (SD)^a^		Participant quotation
	Hospice professionals (*n* = 18)	Family caregivers (*n* = 15)	
1. I don’t want my family member to get addicted to pain medications.	2.94 (0.24)	2.80 (0.41)	“Years ago, my dad was addicted to drugs. I think [this concern] is very important.” — Family Caregiver
2. I can’t tell if my family member is in pain.	2.89 (0.32)	3.00 (0.00)	“Knowing someone could help me recognize the signs and then help me make sure she’s not experiencing pain is very important.” — Family Caregiver
3. I’m worried about potential side effects of pain medications, like drowsiness and constipation.	2.94 (0.24)	2.67 (0.49)	“Constipation and sedation are both possible outcomes of pain medicine, especially if you’re using it injudiciously. But, even when you’re doing your best, it can be a problem.” — Hospice Nurse
4. Pain medications are being used to treat symptoms other than pain, and I don’t understand why.	2.78 (0.43)	2.86 (0.36)	“A lot of these medications for comfort have dual uses … whether it be agitation and nausea or shortness of breath and pain, and I think it’s important that the caregiver understands the different things you can use it for so they feel like they have a lot of tools in their toolbox.” — Hospice Physician
5. I don’t want my hospice team to think I am misusing my family member’s pain medications.	2.56 (0.62)	2.69 (0.48)	“You get things like morphine … and people think like, ‘I don’t want something to go wrong and then it’d be on me.’” — Family Caregiver
6. I don’t want to bother my hospice team or nursing home facility staff with pain management concerns.	2.67 (0.69)	3.00 (0.00)	“[Hospice] need[s] to address that immediately. You don’t want to make the wrong choice [regarding pain medications].” — Family Caregiver
7. I’m worried that pain medications will speed up the dying process.	3.00 (0.00)	2.86 (0.36)	“Me and my mom had a hard time because we’re like, ‘Was this morphine the reason Grandma passed so fast?’ We were really feeling guilty about it.” — Family Caregiver
8. Other than using medications, I’m not sure how to help my family member feel better.	2.83 (0.38)	2.93 (0.27)	“Especially with dementia, it’s like there’s this constantly shifting new normal. [Caregivers often] feel they don’t know what they’re doing. Any tools you can give them to equip them to manage the things that are happening [and] make them feel competent are important.” — Hospice Social Worker

^a^ Importance ratings: 1 = not at all important, 2 = somewhat important, 3 = very important.

### Qualitative

Participants generally indicated high acceptability and reported feeling that EMPOWER-D materials captured prevalent concerns about caring for hospice patients with dementia (see Table 3). Minor suggestions for modifications were also proposed. Qualitative feedback is summarized below, and additional illustrative quotations are provided in Table 3.

#### Concern #1: “I don’t want my family member to get addicted”

All participants viewed this concern as important. Family caregivers expressed worry about providing the appropriate amount of medication to treat pain while avoiding addiction risk and were satisfied with the educational messages as written. All physicians in the sample (*n* = 3) stated they would strengthen the messages by differentiating among addiction, dependence, and tolerance, contending that concerns about addiction primarily stemmed from conflating these concepts. In addition, some hospice professionals suggested that educational messages about addiction should help family caregivers contextualize concerns about patient addiction in light of patients’ terminal status. Rather than adopting a more information-driven approach, a simplified message, such as, “When pain medications are used to treat pain that occurs in hospice, addiction is unlikely,” was recommended.

#### Concern #2: “I can’t tell if my family member is in pain”

This concern was deemed highly important (all family caregivers rated it *very important*), particularly given the communication challenges commonly associated with the later stages of ADRD. Family caregivers noted that the inability to determine whether their family member was in pain was a source of anxiety, stress, and guilt. Some stated that they were taught by their hospice team how to interpret nonverbal cues for pain, and hospice professionals indicated that they typically provide this education proactively. Participants did not suggest substantial changes to the educational messages used. One nurse suggested strengthening the statement “People with dementia can feel pain” by replacing the word “can” with “do.”

#### Concern #3: “I’m worried about side effects”

Concerns about potential side effects of pain medications were deemed important. Constipation was described as especially concerning, whereas drowsiness was cited as common. A number of hospice professionals suggested modifying EMPOWER-D educational messages to include an explanation that drowsiness (which is specifically mentioned in the educational content) can result from disease progression and may not always be attributable to pain medications.

#### Concern #4: “I don’t understand why pain medications are used for other symptoms”

Family caregivers found this topic important to address. A few initially expressed concern that pain medications were used for nonpain symptoms (e.g., “Why [do this]? There are other medications you can use to address [nonpain] issues”). Those who raised concern were satisfied with the EMPOWER-D messages and, after viewing them, expressed their acceptance of pain medications’ use to treat nonpain symptoms. Hospice professionals unanimously found this concern important, with numerous clinicians citing morphine’s dual indication for dyspnea and pain. Two hospice professionals suggested explaining the mechanisms of action that make morphine effective for both pain and dyspnea to strengthen the messages.

#### Concern #5: “I don’t want hospice to think I’m misusing pain medications”

Although still rated high in importance, this concern was considered least important relative to other concerns by hospice professionals. It was also noted as the least clear by both hospice professionals and family caregivers; some participants interpreted “misuse” as over- or under-administration of medication, whereas others interpreted it as medication diversion (the intended meaning). Several hospice professionals indicated that this concern would be crucial to address if raised by family caregivers, suggesting they would interpret it as a “red flag” but that it might not be particularly relevant to all family caregivers. Numerous family caregivers endorsed perceptions of misuse as an important concern, although they reported that they had not necessarily raised it with the hospice team and had intentionally engaged in behaviors (e.g., keeping detailed pain medication logs) intended to quell suspicion.

#### Concern #6: “I don’t want to bother hospice or nursing home staff with pain issues”

All family caregivers rated this concern *very important*, and none suggested changes to the messages. One caregiver recounted their early reaction to being told that hospice providers were available via phone 24/7: “When we first met … our nurse, we were like, ‘We don’t want to bother you … You have a life outside of this.’” Hospice professionals rated this concern as *very* or *somewhat important* to address. They found the relevant educational messages acceptable with no substantial changes, despite generally believing that the teaching points were effectively communicated during routine care.

#### Concern #7: “I’m worried pain medications will speed up the dying process”

Hospice professionals universally rated this item as *very important*, noting it as a common concern among patients’ families. It was also highly valued by family caregivers, some of whom shared helpful phrasing from their hospice team (e.g., “Hospice is not here to speed up the dying process. Hospice is here to make that process comfortable”). Feedback on the corresponding educational messages was mixed, with the consensus that the second teaching point, which extends an offer of support, was stronger than the first, which summarizes clinical research on the topic. One physician opined that the first educational message included weak verbiage (i.e., “There is no evidence” that pain medications hasten death) and recommended, instead, more definitive verbiage (i.e., “Pain medication does not hasten death”).

#### Concern #8: “Other than using medications, I’m unsure how to manage pain”

Family caregivers’ responses to this item were decidedly positive both quantitatively and qualitatively. In addition to rating the concern as important, several family caregivers cited nonpharmacological interventions (e.g., music therapy, massage) as beneficial in ameliorating pain. Hospice professionals were also enthusiastic about this item and offered additional examples that could be included in the educational messages. Two chaplains specifically recommended spiritual interventions (e.g., pastoral presence, prayer) to be included among the examples of nonpharmacological pain responses.

## Discussion

Building from a developing evidence base,^16,17^ the current study elicited feedback from hospice professionals and family caregivers of hospice patients with dementia about the educational content covered in the EMPOWER-D intervention materials. Participants indicated that the EMPOWER-D materials addressed common pain concerns that were both family-centered and relevant to clinical dementia care. Underscored by the parallel increases in US population aging,^25^ hospice utilization,^26^ and ADRD prevalence,^27^ these results lend preliminary support for EMPOWER-D as a promising means to improve pain management for hospice patients with dementia.

Quantitative results indicated that participants generally found the EMPOWER-D educational content to be acceptable. Although family caregivers’ and hospice professionals’ ratings were overwhelmingly similar, the largest difference observed between the two pertained to the concern about family members not wanting to bother the hospice team or nursing home facility staff with pain management concerns. All family caregivers rated this concern as *very important*, whereas some hospice professionals rated it as *somewhat important*. This discrepancy suggests that some hospice professionals may underestimate family caregivers’ reluctance to reach out with pain concerns, which is concerning, as family caregivers function as key partners with hospice professionals in the provision of patient care.^28,29^ Thus, amid ongoing pain assessment challenges in dementia,^20,21^ the presence of additional, remediable barriers, potentially culminating in underreporting symptom burden, poses a substantial threat to the provision of optimal patient care.

Although qualitative findings largely supported the high acceptability demonstrated in the quantitative results, recommendations were provided to make educational messages more precise and definitive for family caregivers. Importantly, caution should be exercised in crafting more definitive language. Oversimplified messages, such as “Pain medication does not hasten death” or “Opioids never cause addiction when used as prescribed,” could potentially convey information that is inaccurate—particularly in nonhospice contexts—and could undermine family caregivers’ trust. In other instances, recommended revisions (e.g., changing “People with dementia can feel pain” to “People with dementia do feel pain”) may enhance the person-centeredness of the EMPOWER-D educational materials and increase their overall potential benefit.

Results should be considered alongside extant limitations. Despite affording increased depth to the current study’s predominantly qualitative objective, our use of purposive sampling means that these results do not generalize beyond the current sample. Relatedly, owing to insufficient statistical power, we were unable to assess mean differences between family caregivers and hospice professionals in inferential frameworks. Results are also limited by risk of self-report bias brought on by the interviewer-facilitated data collection format.

Combined with the documented efficacy of the original EMPOWER intervention,^16^ these findings support continued development and future testing of EMPOWER-D.^30^ Primary targets for next steps in future research include assessing EMPOWER-D’s feasibility and conducting pilot testing. Such efforts could pave the way for larger, more definitive clinical trials to determine EMPOWER-D’s efficacy in reducing pain experienced by hospice patients with dementia and addressing concerns about pain and pain medications reported by these patients’ family caregivers.

## Abbreviations Used

ADRD: Alzheimer's disease or a related dementia
EMPOWER: Effective Management of Pain: Overcoming Worries to Enable Relief
EMPOWER-D: Effective Management of Pain: Overcoming Worries to Enable Relief-Dementia
